# Investigating hesperetin nanocrystals with tailor-made sizes for the prevention and treatment of Alzheimer’s disease

**DOI:** 10.1007/s13346-020-00888-0

**Published:** 2021-01-12

**Authors:** Pascal-L. Stahr, Rekha Grewal, Gunter P. Eckert, Cornelia M. Keck

**Affiliations:** 1 Department of Pharmaceutics and Biopharmaceutics, Philipps-Universität, Robert-Koch-Str. 4, 35037 Marburg, Germany; 2 Institute of Nutritional Sciences, Justus-Liebig Universität, Wilhelmstr. 20, 35392 Gießen, Germany

**Keywords:** Hesperetin, Nanocrystals, Zeta potential, Alzheimer’s disease, Antioxidant

## Abstract

**Abstract:**

Poor aqueous solubility of drug substances is associated with poor bioavailability and thus hampers the effective use of many potent active pharmaceutical ingredients. Various strategies to overcome poor solubility are available, whereby drug nanocrystals represent one of the most powerful formulation strategies to enhance the kinetic solubility and dissolution rate of poorly soluble drugs. Nanocrystals are simply obtained by milling large-sized drug powders to sizes < 1 µm. The so obtained nanocrystals possess an increased dissolution rate and kinetic solubility when compared with larger-sized bulk material. The aim of this study was to produce differently sized hesperetin nanocrystals and to investigate the influence of nanocrystal size on the bioefficacy of the natural antioxidant hesperetin in two cell culture models for the prevention and treatment of Alzheimer’s disease. Results showed that the testing of poorly soluble compounds is challenging and requires incredibly careful characterization. Reasons for this are possible changes of the formulations in cell culture media which can occur due to various reasons. If the changes are not considered, results obtained can be misleading and even lead to a false interpretation of the results obtained. Besides, results demonstrate the increase in dissolution rate with decreasing particle size that is especially pronounced with particle sizes < 200 nm. Data also provide clear evidence that smaller nanocrystals with higher kinetic solubility possess higher antioxidant capacity. This results in lower amounts of free radicals in the cell culture models, suggesting that hesperetin nanocrystals, that improve the poor aqueous solubility of hesperetin, are promising for the prevention and treatment of Alzheimer’s disease.

**Graphical abstract:**

## Introduction

Hesperetin is a secondary plant metabolite that is found in various plants. It is well-known for its antioxidant properties and thus possesses a great potential to be used as therapeutic active to treat or prevent oxidative stress-related diseases. Especially neurodegenerative disorders, that cannot be treated or prevented so far, are of high interest [1, 2]. However, the major hurdle that needs to be overcome is its poor aqueous solubility, which results in poor bioavailability [3, 4]. Consequently, a formulation that increases the solubility and bioavailability of hesperetin is a prerequisite for the use of hesperetin as therapeutic active compound. A simple solution to overcome poor aqueous solubility is the use of organic solvents in which the active pharmaceutical ingredient (API) can be dissolved. This strategy is frequently used in cell culture models, where solvents, e.g. DMSO or alcohols, are typically used to dissolve the APIs. This straight forward strategy allows for an early determination of pharmacological and toxicological effects of such compounds in-vitro, but—due to the toxicity and regulatory issues of the organic solvents—it cannot be used as universal formulation strategy for poorly soluble actives in in-vivo applications. Hence, other formulation principles are required to apply poorly soluble APIs in-vivo [5].

To date, the most well-known formulation principle to increase the dissolution rate and the solubility of poorly soluble APIs is the production of drug nanocrystals [6–11]. Nanocrystals are composed of 100% API and possess sizes below 1 µm. Various methods are available to produce nanocrystals from large-sized bulk material. Most prominent production techniques involve precipitation methods or different milling methods, e.g. bead milling, high-pressure homogenization or combinations of these methods. Independent on the method used to obtain nanocrystals, size reduction results in an increase in surface area and thus—based on the Noyes-Whitney equation—an increase in dissolution rate. Surface reduction also results in an increase in the curvature radius and thus—based on the Kelvin equation—leads to an increase in the kinetic solubility [8–11]. Recent studies could already demonstrate that hesperetin can be transferred into nanocrystals [3] and very recent studies by Zare and co-workers already showed the great potential of this formulation principle to treat neurodegenerative disorders, e.g. Alzheimer’s disease (AD) or autism spectrum disorders (ASDs) in animal models [1, 2]. Even though the results prove the great potential of the nanocrystals, it remains unclear what size should be preferred for the formulation of hesperetin. In general, it is assumed that the smallest size is the most suitable approach, because solubility and dissolution rate increase with decreasing size. On the other hand, one needs to consider that the production of small-sized nanocrystals (< 200 nm) requires more efforts, i.e. time and costs, than the production of larger-sized nanocrystals. Therefore—especially when considering the formulation of nanocrystals in large scale—it is highly interesting to gain more knowledge of the influence of nanocrystal size on the biological effects and to determine the most suitable size, which enables both—excellent biological efficacy and the possibility to produce these formulations fast and cost-efficiently in large scale.

The aim of this study was to address this issue and to gain more detailed information about the influence of size on the biological effects of hesperetin nanocrystals. In the first step of the study, hesperetin nanocrystals of different sizes were produced by applying different formulation approaches for the production of the hesperetin nanocrystals [12]. In the next step the physical-chemical properties of the nanocrystals, i.e. size, zeta potential, crystallinity, and dissolution behaviour, were determined and compared with large-sized bulk material. Finally, the biological efficacy, i.e. antioxidant capacity (AOC) and adenosine triphosphate (ATP) production, was determined in-situ and in an established cell model of the initial phase of sporadic AD, respectively [13]. Hesperetin dissolved in ethanol served as control.

## Materials and methods

### Materials

Hesperetin was purchased from Exquim S.A. (Spain). Alkyl polyglycosid (Plantacare 2000 UP®) was used as stabilizer [12, 14] and was a kind gift from BASF (Germany). All formulations contained 5% (w/w) hesperetin and 1% (w/w) stabilizer in purified water. Purified water was obtained from a PURELAB Flex 2 (ELGA LabWater & Veolia, Germany). All other analytical chemicals were of analytical grade and were used as received.

### Methods

#### Production of nanocrystals

**Fig. 1:**
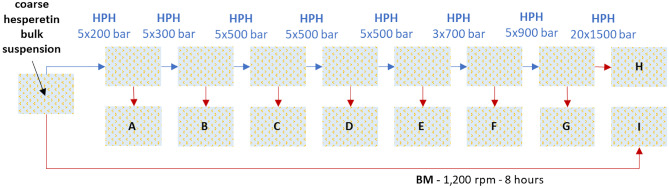
Scheme of production of the differently sized nanosuspensions

The production of nanocrystals can be performed by using various techniques, i.e. rotor-stator high speed stirring (HSS), high-pressure homogenization (HPH), and bead milling (BM). Each method uses different principles of size reduction, and thus, the different techniques can be used to yield differently sized nanocrystals [12, 15–19]. HPH with and without the combination of HSS is a fast and high energy process that leads—depending on production parameters and the properties of the material—to nanocrystals with sizes of about 400–800 nm [12, 20, 21]. Bead milling is a low energy process, requires long milling times, and typically leads to small-sized nanocrystals with sizes of around 200 nm and a narrow size distribution [19, 22]. Small-scale wet bead milling (BM) is a new, improved process developed by Romero et al., which can be used for the production of ultra-small batch sizes and is thus highly suitable for early phases in drug formulation development [23]. As this study aimed for the production of both small-sized and larger-sized drug nanocrystals, HPH and BM were applied for the production of the differently sized nanocrystals. Prior to HPH or BM coarse hesperetin suspensions containing 5% (w/w) hesperetin and 1% (w/w) stabilizer in purified water were prepared. The coarse suspensions were subsequently subjected to HPH or BM, respectively. HPH was performed with a Micron LAB 40 (GEA, Germany) by applying different numbers of homogenization cycles and pressures to the formulations (Fig. 1). Small-scale BM was performed by placing the coarse suspension in a 2-ml glass vial with three stirring bars and yttria stabilized zircon oxide milling beads (diameter 1 mm, Retsch, Germany) with a suspension to bead ratio of 1:1 (V/V). All vials were stirred on a magnetic stirring plate (IKA RCT standard, Germany) at 1,200 rpm in ice water for 8 h [23].

#### Characterization of nanocrystals

The physicochemical properties of the nanosuspensions were characterized regarding size, zeta potential, crystalline state, and dissolution behaviour.

#### Size characterization

Particle size analysis was performed by three different and independent techniques. The mean particle size was determined by using dynamic light scattering (DLS), also known as photon correlation spectroscopy (PCS), by using a ZetaSizer NanoZS (Malvern-Panalytical, Germany). The results are expressed as hydrodynamic diameter (z-average) and polydispersity index (PdI), which is a measure of the width of the size distribution. A narrow size distribution is achieved with PdI values < 0.2. Possible larger particles, i.e. sizes > 6 µm, cannot be detected by DLS measurements. Therefore, to allow for a thorough characterization of the samples, laser diffraction (LD) with a measuring range to up to 3500 µm was employed to detect possible larger particles within the small-sized main population [24]. LD-measurements were performed by using a Mastersizer 3000 (Malvern-Panalytical, Germany). Analysis was performed by using the Mie theory with real and imaginary refractive indices of 1.59 and 0.01, respectively [25]. Results are expressed as median volume weighted diameters *d*(*v*) 0.50, 0.90, 0.95, and 0.99. The number given, e.g. *d*(*v*) 0.50, indicates that 50% of the volume of the particles possess a size being equal or below the given value. To further prove the results obtained from DLS and LD, light microscopy was used [24, 26]. Visual observation, i.e. determination of possible agglomerates or larger crystals that remained untriturated during the homogenization step, was performed by using an Olympus BX53 light microscope (Olympus Cooperation, Japan), equipped with an Olympus SC50 CMOS colour camera (Olympus soft imaging solutions GmbH, Germany).

#### Zeta potential analysis

Zeta potentials (ZP) were analysed by using a ZetaSizer NanoZS (Malvern-Panalytical, Germany). The ZP was determined in purified water which was adjusted to a conductivity of 50 µS/cm with sodium chloride solution. In addition, for a more detailed information on the stabilizing mechanisms, zeta potentials were also analysed in the original dispersion medium (surfactant solution) and the cell culture medium (Table 1).

**Table 1:** Overview of dispersion media used for zeta potential analysis

Dispersion medium	Composition
Water	Purified water adjusted to a conductivity of 50 µS/cm with NaCl
Original dispersion medium (OM)	Surfactant solution = Plantacare 2000 UP 1% (w/w) in purified water
Cell culture medium (CM)	DMEM and foetal calf serum in a ratio 9:1 (v/v)

#### Determination of crystalline state

The crystalline state was determined by X-ray diffraction (XRD) by using a X’Pert Pro MPD powder X-ray diffractometer with a PIXcel detector (Panalytical, Germany) and a CoKα radiation source (*λ* = 1.54187 Å). Samples were measured to CoKα radiation (40 kV, 35 mA) at a scanning rate of 2.4°/min between 10 and 70° 2*θ* with a step size of 0.039° 2*θ*. To enhance the viscosity of the liquid suspensions, prior the measurements, locust beam gum (3% (w/w)) was added to the aqueous suspensions. This was done to avoid the need for drying of the samples prior to analysis, which in turn could induce crystallization or crystal growth and thus lead to changes in the crystalline structure of the original suspensions [27].

#### Solubility of hesperetin bulk material

Based on literature the solubility of hesperetin is 273 mg/L [28, 29]. However, besides this value, also other and much lower values are reported in the literature [30–33]. The reasons for the different values are not clear but indicate the necessity to determine the solubility of each batch individually. The saturation solubility of the bulk material was therefore determined by adding an excess of hesperetin (about 800 mg) and 15.0 ml purified water into a 30-ml universal glass vial (VWR International GmbH, Germany). The vial was sealed with the screw cap and placed on a magnetic stirring plate (IKA RCT standard, Germany) for 24 h in a climate chamber at 37 °C. After stirring for 24 h at 800 rpm, the suspension was rested for about 1 h to allow sedimentation of larger particles. Subsequently, 2 ml of the coarse suspension were withdrawn and filtered through 0.22 µm filters. Subsequently, the filtrate was centrifuged for 30 min at 14,000 rpm (Eppendorf Centrifuge 5418, Eppendorf, Germany), and the content was quantified by high performance liquid chromatography (HPLC). The experiment was run in triplicate, and from each vial, three independent samples were drawn to exclude possible defects between the test conditions and filters.

#### Dissolution behaviour

Dissolution studies were performed to investigate the dissolution velocity and the aqueous solubility of the differently sized nanosuspensions. For this an USP-II paddle apparatus (Pharma Test PTWS 120D, Germany) at 37 ± 0.5 °C and 100 rpm in 900 ml of purified water was used. Tests were performed in non-sink conditions, i.e. in saturated medium. The amount of added hesperetin was 24.57 mg for each formulation, which resulted in a final concentration of 27.3 mg hesperetin/L. The exact volume required for this was different for each formulation, because due to the different production methods, the hesperetin content was slightly different in the different final formulations. Reasons for this are for example the adsorption of some hesperetin to the beads in the bead milling process or sedimentation of larger—not yet homogenized—particles to the bottom of the product container during the discontinuous high-pressure homogenization process used in this study. The exactly required volume of each hesperetin formulation was therefore calculated after the hesperetin content was analysed by HPLC analysis for each formulation individually. At predetermined time intervals samples of 1 ml were drawn and the volume collected was re-filled with equal volumes of fresh medium. To ensure the complete retention of particles in the collected samples, all samples were filtered directly through 0.22 µm filters, and centrifuged for 30 min at 14,000 rpm (Eppendorf Centrifuge 5418, Eppendorf, Germany), immediately after drawing the samples. The amount of dissolved hesperetin was determined by HPLC analysis. Experiments were performed in triplicate, and the results are presented as mean ± SD.

#### HPLC analysis

Drug concentrations were determined by high performance liquid chromatography (HPLC) using an Agilent Infinity II 1260 (Germany) that was equipped with a G7111A 1260 Quat Pump VL, a G7129A 1260 sampler, and an Agilent Poroshell 120 EC-C18, 4.6 × 50 nm, 2.7 μm analytic column. A solvent mixture containing methanol, water, and acetic acid (50:48:2 (V/V/V)) as mobile phase was used under isocratic elution. The flow rate was set at 0.45 ml/min and the temperature at 45 °C. The injection volume was 5 μl, and the expected retention time was 2.8 min. A diode array detector (G7117C 1260 DAD HS) was used as UV detector and operated at 288 nm. The calibration curves were performed every day of the analysis, and the linearity was confirmed from 0.98 to 125 μg/ml (*R*^2^ = 0.9999). For all measurements, only this concentration range was used for calculating the results. All measurements were carried out in triplicate.

#### Determination of antioxidant capacity

#### DPPH assay

The antioxidant capacity (AOC) was assessed by using the DPPH assay [34]. DPPH (1,1-diphenyl-2-picryl-hydrazyl, Sigma-Aldrich, Germany) is a free radical that can be reduced by antioxidants. Upon reduction, the colour of the free radical changes, and thus, the amount of reduced DPPH can be accessed via UV-Vis spectroscopy. The DPPH assay is performed for different concentrations of the antioxidant, which enables the determination of the IC 50 that is the concentration needed to scavenge 50% of the free radical (DPPH). Consequently, low IC 50 values represent a high AOC and vice versa.

In this study, the IC 50 was determined at different time points and in different dispersion media, i.e. purified water and ethanol, respectively. Tests were performed by adding 100 µl of the samples containing different concentrations of the nanocrystals to 100 µl of a 0.3 mM ethanolic solution of DPPH. After incubation (5 min, 15 min, 20 min, 30 min and 45 min) in the dark, the absorbance was measured by a UV-VIS plate reader (Mulitskan GO, Thermo scientific, Germany) at 517 nm. The inhibition activity (inhibition %) was calculated as: $\rm{inhibition}\left[\%\right]=\left({1-} {{\rm{A}}_{\rm{sample}}}/{\rm{A}}_{0}\right) \cdot 100$, where *A*_sample_ is the absorbance of the sample and *A*_0_ is the absorbance of the control (DPPH solution). The resulting function of inhibition against concentration was used to calculate the IC 50 value (µg/ml). Besides the determination of the IC 50 for the nanocrystals, also, the IC 50 of the bulk material and an ethanolic hesperetin solution was analysed and used as control.

#### Determination of bioenergetics in Alzheimer cell culture model

For the determination of the biological activity, the formulations were tested in a SH-SY5Y AßPP_wt_ cell culture model [13]. SH-SY5Y cells are stably transfected with DNA constructs containing the entire coding region of the human beta-amyloid precursor protein (AßPP; AßPP 695). Due to this SH-SY5Y AßPP_wt_ cells show typical symptoms of early AD, i.e. slightly elevated beta-amyloid (Aß) levels, moderately decreased ATP levels, impaired mitochondrial membrane potential and decreased mitochondrial respiration [13]. As a result, this leads to oxidative stress in the cells. Hence, if potent antioxidants are added to the cells, the vitality of the cells increases which results in an increase in ATP levels. Therefore—by determining the changes in ATP levels—this cell culture model can be used to pre-evaluate the efficacy of an antioxidative formulation for the prevention of early AD. Moreover, predicting the efficacy of a formulation to treat later phases of AD can also be simulated by adding rotenone, which inhibits the complex I, whose dysfunction is one of the most prominent alteration in late AD [13].

For the experiments, cells were cultured in Dulbecco’s Modified Eagle Medium (DMEM) supplemented with 10% heat-inactivated foetal calf serum, 0.3 mg/ml hygromycin, 60 units/ml penicillin, 60 µg/ml streptomycin, MEM Vitamin solution, MEM Non-Essential Amino Acids, and 1 mM sodium pyruvate at 37 °C in a humidified incubator containing 5% CO_2_. For the determination of the ATP-levels 2 × 10^4^ cells were seeded in a white walled 96 well and grown for 24 h. Cells were incubated with the respective hesperetin concentrations (0.01–10 µM) for 24 h or pre-incubated with hesperetin for 1 h and insulted with Rotenone (25 µM) for 24 h. Subsequently, ATP levels were determined by using the ViaLight® Plus bioluminescence kit (Lonza, Walkersville, USA), which is based on the production of light from ATP and luciferin in the presence of luciferase. The assay was performed according to the manufacturer’s instructions. The emitted light is linearly to the ATP concentration and was recorded using a Multilabel Plate Reader (Victor2 1420 multilabel counter, Perkin Elmer, Germany).

#### Statistical analysis

Statistical analysis was performed with JASP software (version 0.13.1) [35]. Probability values (*p* values) < 0.05 were considered to indicate significant differences.

## Results and discussion

### Production and characterization of nanocrystals

**Fig. 2:**
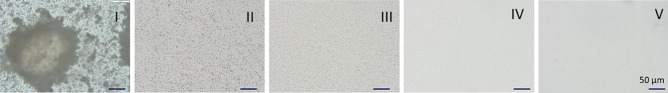
Microscopic images of hesperetin suspensions. I: bulk material, II: after HPH with low pressure, III: after HPH with medium pressure, IV: after HPH with high pressure, V: after bead milling (magnification × 400)

The application of different production techniques and production parameters yielded hesperetin nanocrystals with different sizes. The sizes ranged between approximately 200 and 800 nm (Table 2). As expected, the smallest particles and the narrowest size distributions were obtained with the BM process (formulation I). Larger sizes and broader size distributions were obtained with the HPH process (formulations A-H). The application of very low pressures (formulation A) did not result in submicron particles, but already the application of slightly more homogenization cycles and slightly higher homogenization pressures (formulation B) led to nanocrystals with sizes of about 800 nm. Medium pressures of 500 bar (formulations C-E) yielded sizes between approximately 520–660 nm and a further increase in pressure to 700 and 900 bar could further reduce the size to about 500 and 400 nm (formulations F and G). High-pressure homogenization with 20 cycles at 1500 bar (formulation H) did not further decrease the mean particle size of the nanocrystals and led to nanocrystals with sizes being similar to the sizes of formulation G, which was only homogenized by applying 900 bar. Also, microscopic analysis and LD data confirmed a pronounced decrease in size and a reduction of larger-sized microcrystals with increasing homogenization pressures and numbers. However, also, LD did not detect any differences in size between formulation G and H (Table 2; Fig. 2), indicating that HPH at medium pressures is sufficient for the production of hesperetin nanocrystals. This may be due to relatively poor hardness of hesperetin, as has previously also been reported for other flavonoids [36].

**Table 2:** Particle size of hesperetin nanocrystals. I: LD data (median volume diameters (d(v)0.5 – d(v)0.95). II: PCS data (z-average and PdI)

**I**	**particle size - LD data [µm]**											
	**d(v)0.5**			**d(v)0.90**			**d(v)0.95**			**d(v)0.99**		
**bulk**	8.5	±	0.02	24.9	±	0.04	36.1	±	0.11	48.5	±	0.04
**A**	4.4	±	0.03	10.5	±	0.03	12.5	±	0.04	16.0	±	0.06
**B**	2.3	±	0.03	6.6	±	0.01	8.1	±	0.04	10.8	±	0.14
**C**	1.2	±	0.04	4.5	±	0.03	5.7	±	0.01	8.2	±	0.02
**D**	0.6	±	0.02	3.2	±	0.04	4.2	±	0.10	6.7	±	1.07
**E**	0.8	±	0.04	3.0	±	0.03	3.7	±	0.03	5.3	±	0.03
**F**	0.9	±	0.01	2.8	±	0.05	3.5	±	0.08	5.5	±	0.62
**G**	0.6	±	0.01	2.0	±	0.01	2.5	±	0.02	3.4	±	0.02
**H**	0.5	±	0.01	2.0	±	0.01	2.4	±	0.01	3.4	±	1.01
**I**	0.1	±	0.01	0.3	±	0.00	0.4	±	0.01	0.6	±	0.07
**II**	**particle size - DLS data**											
	**z-average [nm]**						**PdI**					
**A**	-						-					
**B**	778			±	148		0.51			±	0.12	
**C**	656			±	98		0.55			±	0.07	
**D**	549			±	82		0.5			±	0.09	
**E**	522			±	79		0.42			±	0.08	
**F**	495			±	28		0.44			±	0.10	
**G**	407			±	18		0.36			±	0.06	
**H**	393			±	15		0.35			±	0.06	
**I**	172			±	2		0.16			±	0.04

**Fig. 3:**
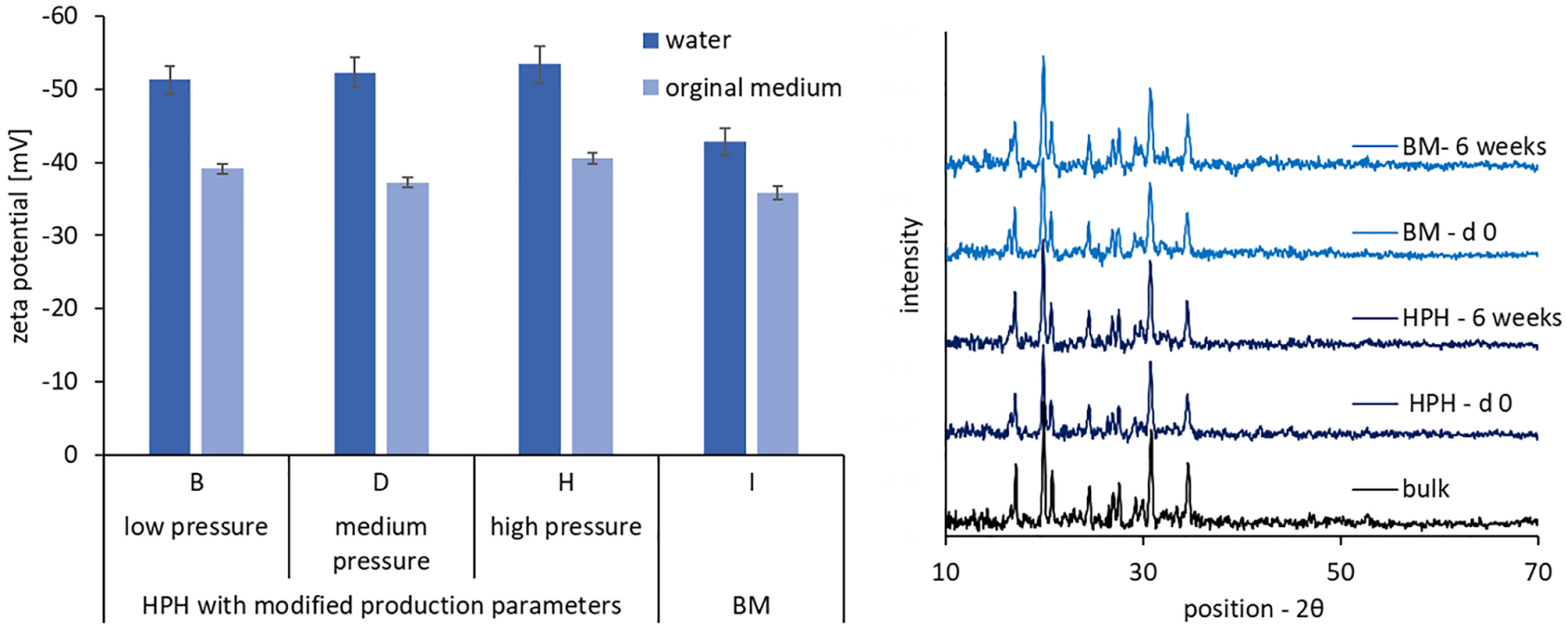
Determination of zeta potentials in water and original dispersion medium (left) and determination of crystalline state by X-ray diffraction (right)

The zeta potential is related to the surface charge of nanoparticles and is typically used to predict the physical stability of colloidal dispersions [37]. In this study Plantacare 2000 was used as stabilizer. It is a non-ionic surfactant but is known to form electrostatically charged micelles [38], thus leading to a combination of steric and electrostatic stabilization mechanisms and relatively high zeta potentials [39]. Also, in this study high zeta potentials > |30 mV| were obtained for both media, indicating excellent electrostatic stabilization of the nanocrystals (Fig. 3, left).

However, it was also observed that the ZP of the BM nanocrystals in water was slightly lower than the ZP of the HPH processed nanocrystals when analysed in water, albeit no differences in ZP were found when the ZP was analysed in original dispersion media. Analysis of the ZP in original dispersion media is believed to represent the real charge of the particles during storage and is important to predict the physical stability of the particles during storage. Analysis in water “dilutes” the particles and surfactant being only tightly bound to the surface of the particles can desorb. Thus, by comparing the ZP results obtained in water and original dispersion medium, it is possible to judge if the surfactant layer is tightly bound to the surface of the particles or not. The smaller the difference, the more tightly bound is the surfactant to the surface of the particle, and thus, the more pronounced is the stabilization efficacy of the surfactant [25].

In this study, the ZP were only different when the ZP was analysed in water, indicating that the surface of the nanocrystals produced by BM possesses different properties than the nanocrystals produced by HPH. One possible explanation for this observation would be the formation of an amorphous hesperetin layer around the crystalline core of the nanocrystals due to the milling, which was already reported previously for various other APIs [40]. To prove or disprove this theory, X-ray diffraction patterns were obtained for all formulations (Fig. 3 right). Results did not show any differences between the different nanocrystals. Hence, it can be assumed that the differences in ZP are not due to changes in crystalline state but are related to other circumstances. Therefore, the most likely explanation for the differences in ZP would be the different surface properties of the nanocrystals, which are related to the different diminution principles. The HPH process mainly uses cavitation forces for the diminution of the particles, whereas BM uses mainly shear forces [16, 18, 22]. Hence, depending on the diminution technique used, the crystalline lattice of the crystals will break in different directions, thus leading to different newly created surfaces, with different surface properties, which in consequence, can cause different ZP values [36].

### Determination of solubility and dissolution behaviour

**Fig. 4:**
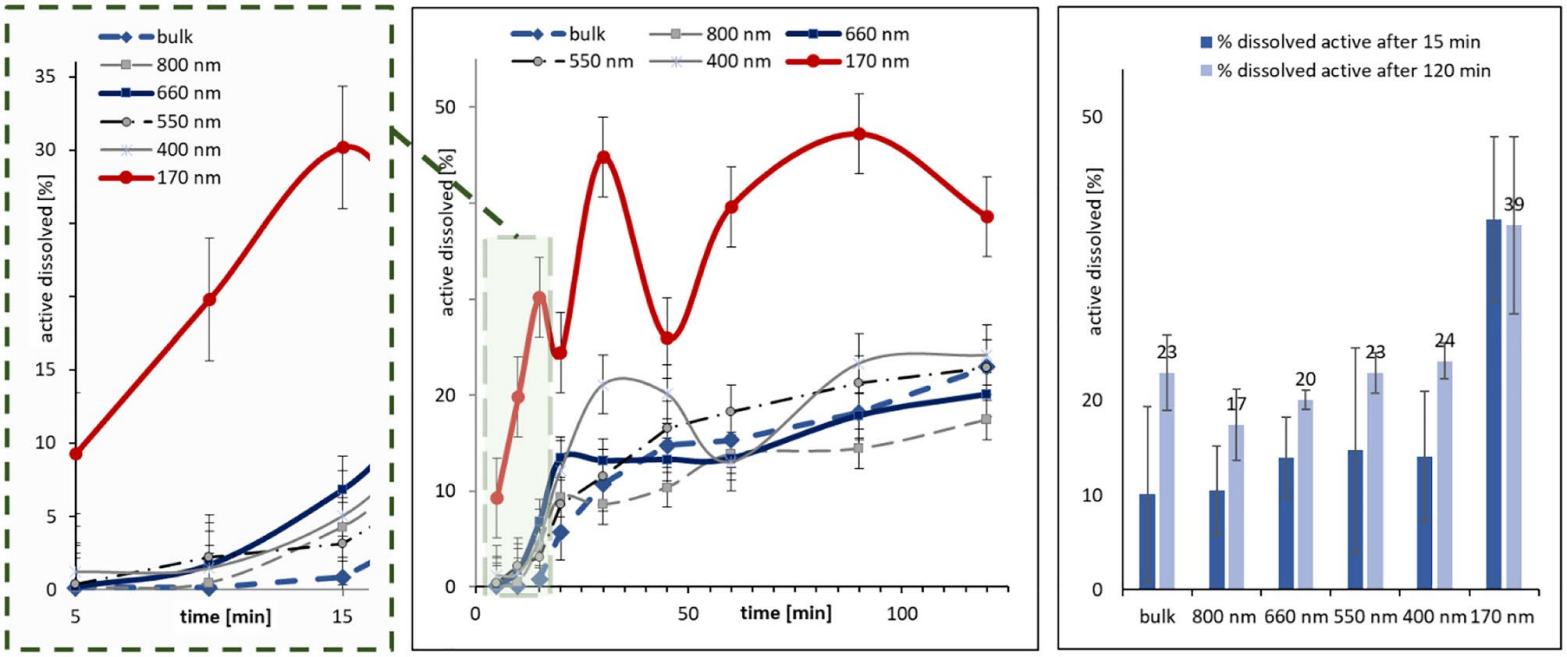
Dissolution behaviour of differently sized hesperetin nanocrystals in comparison to large-sized bulk material. Left: Dissolution profiles within 15 min, middle: Dissolution profiles within 2 h, right: Solubility of hesperetin from differently sized nanocrystals after 15 and 120 min

The solubility of the hesperetin bulk material in water at 37 °C was determined to be 26.4 µg/mL. With this it was well in between the values that were previously published [28, 32]. In the next step of this study, the kinetic solubility of the differently sized particles was determined, and the results were compared with the large-sized bulk material (Fig. 4). Results revealed no significant differences in dissolution velocity and kinetic solubility between bulk material and nanocrystals with sizes > 500 nm and only small differences were found in the total amounts of dissolved active. However, especially in the beginning, the dissolution velocity was higher than that of the larger-sized bulk material (Fig. 4, left). A significant increase in both dissolution velocity and solubility was determined for the nanocrystals with sizes < 200 nm. During the first 15 min of the dissolution experiment, the amount of dissolved active was at least 5 times higher for the small-sized nanocrystals (170 nm), when compared with the larger-sized crystals (Fig. 4, left). However, after about 15 min a decrease in the amount of dissolved active was observed, which increased again during the next minutes of the experiments and subsequently decreased again (Fig. 4, middle).

The observation can be explained by the increased kinetic solubility of the small-sized nanocrystals, which results in a pronounced dissolution of the active from the nanocrystals at the beginning of the experiment. The fast dissolution of the active leads to a supersaturated solution of hesperetin in which the amount of dissolved active is above the thermodynamic solubility of the active. Consequently, as this supersaturated solution is not thermodynamically stable, re-crystallization of the supersaturated active is highly likely to occur. Re-crystallization leads to a decrease in the amount of dissolved active and thus explains the observation done for the nanocrystals with sizes < 200 nm. The same effects—to a much less pronounced extent—were also observed for the nanocrystals with sizes of 400 nm but were not observed for the nanocrystals with sizes > 500 nm. These results demonstrate a size-dependent increase in dissolution rate and kinetic solubility. However, these benefits may be compromised due re-crystallization of the active to form supersaturated solutions. Nevertheless, even though some re-crystallization was observed for the small-sized nanocrystals, their all-over kinetic solubility was about twofold higher when compared with bulk material and the larger-sized nanocrystals (Fig. 4, right). Due to the small and almost negligible differences between the larger-sized formulations, only one formulation from the group of larger-sized nanocrystals (660 nm) was selected for all further experiments. This formulation, together with the small-sized nanocrystals (170 nm) and the bulk material, was used for the determination of the antioxidant capacity, which was determined in-vitro and in the Alzheimer cell culture model.

### Determination of antioxidant capacity

#### Determination of antioxidant capacity in vitro

**Fig. 5:**
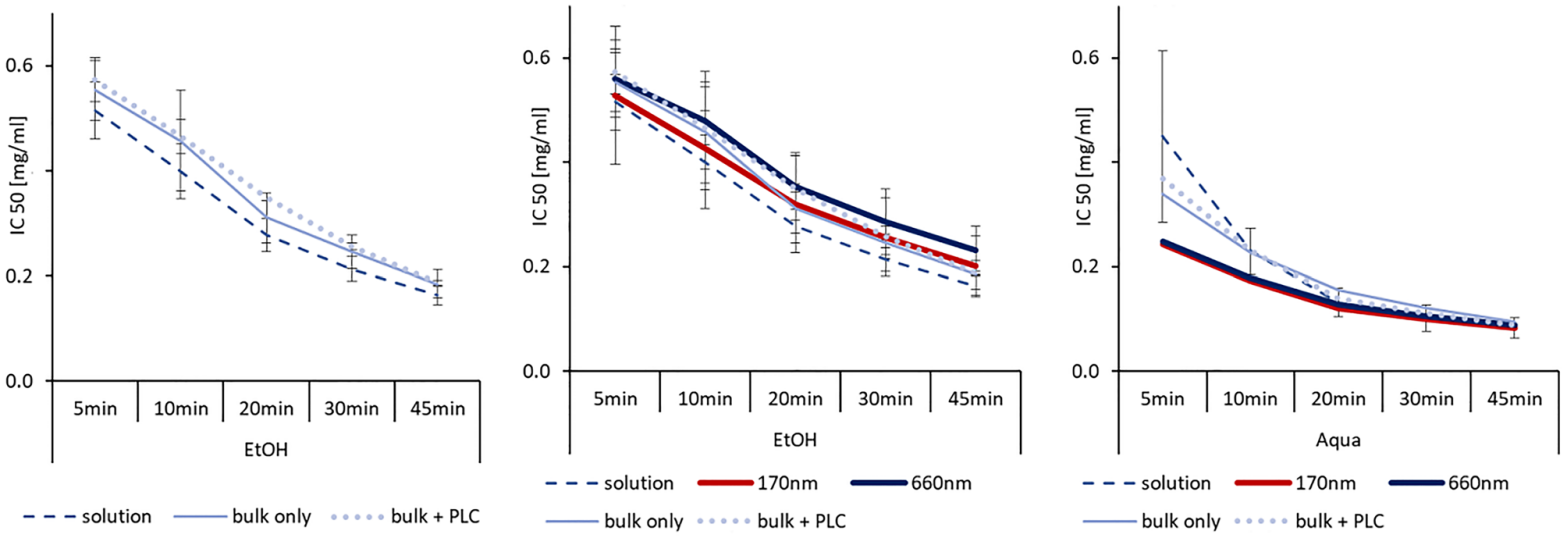
Determination of antioxidant capacity by DPPH assay. Left—DPPH assay of ethanolic hesperetin solution vs. hesperetin bulk material with and without stabilizer (PLC = Plantacare 2000) in ethanolic dispersion medium; middle—DPPH assay of ethanolic hesperetin solution and bulk material vrs. hesperetin nanocrystals in ethanolic dispersion medium; right—DPPH assay of ethanolic hesperetin solution and bulk material versus hesperetin nanocrystals in aqueous dispersion medium

The AOC was determined in-vitro by assessing the amount needed to scavenge 50% of the free radical DPPH (Fig. 5). The results allowed the determination of the IC 50 value, where low values indicated a high AOC. The IC 50—which is also a surrogate for the biological AOC of a formulation—is related to the amount of dissolved active, because only dissolved active, i.e. discrete molecules, can react with their specific target. Today, in most biological assays, poor solubility is overcome by simply dissolving the API in organic solvents, e.g. DMSO or alcohols. Therefore, to allow for a discrimination of the AOC between a standard formulation (solution in organic solvent) and the nanosuspensions, in this study not only the selected suspensions but also an ethanolic hesperetin solution was used for the determination of the IC 50.

In the first step, the IC 50 of bulk material was compared with the IC 50 values of the ethanolic solution (Fig. 5, left). Tests were performed in ethanol and revealed a higher IC 50 (lower AOC) for the bulk material. Over time the AOC increased for both formulations, and after 45 min almost no differences in AOC were determined between both formulations. Results were expected and can be explained as follows: The DPPH assay is a chemical reaction. Hence, the number of free radicals scavenged by the added antioxidant increases over time, which results in a decrease in IC 50 value over time. The effects can be seen for the ethanolic hesperetin solution. In contrast, the hesperetin suspension contains less dissolved molecules; thus, at the beginning, less DPPH is scavenged, and thus, when compared with the solution, higher IC 50 values are observed. Over time hesperetin dissolves, leading to a decrease of the IC 50. After 45 min of reaction time probably all hesperetin particles are dissolved, but the time for reaction was less, and thus, the IC 50 values are still slightly higher than the values obtained for the ethanolic solution. The test was also performed for bulk material containing surfactant (Fig. 5, left), and it was found that the addition of surfactant delayed the decrease in IC 50 over time. The reason for this might be the encapsulation of dissolved active into micelles, which reduced the amount of free active molecules, thus resulting in the observed decrease in the IC 50 over time.

In the next step, the IC 50 for the nanocrystals (170 nm and 660 nm) was determined (Fig. 5, middle). In the beginning, the small-sized nanocrystals were found to possess similar IC 50 values than the solution, whereas the larger-sized nanocrystals possessed IC 50 values being similar to the values obtained for the bulk material. As discussed earlier, the IC 50 value is related to the amount of dissolved active, and thus, the results obtained correlated nicely to the solubility data obtained in the previous part of the study (c.f. 3.2). With increasing time, the IC 50 values of the nanocrystals decreased slower than the values for the bulk material and the solution. Reasons for this might be the surfactant and the encapsulation of the active into micelles and/or agglomeration of the nanocrystals in the ethanolic environment, leading to a reduced solubility of hesperetin. However, the assessment of the IC 50 in the ethanolic dispersion medium was performed to gain details about the reaction mechanism. However, this test environment does not represent the environment used in in-vivo studies. Therefore, in the next step, the IC 50 was determined in aqueous media (Fig. 5, right). Results obtained were almost opposite to the results obtained in ethanolic medium. At the beginning, the highest AOC was found for the nanocrystals, the second-best AOC was found for the bulk material, and the lowest AOC (highest IC 50 value) was found for the ethanolic solution. Over time, the differences became smaller and cancelled out after 45 min of reaction time. The differences between bulk material and nanocrystals were not significant. However, the trend observed is reasonable and can be explained with the increased dissolution velocity and improved kinetic solubility of the nanocrystals (c.f. 3.2).

**Fig. 6:**
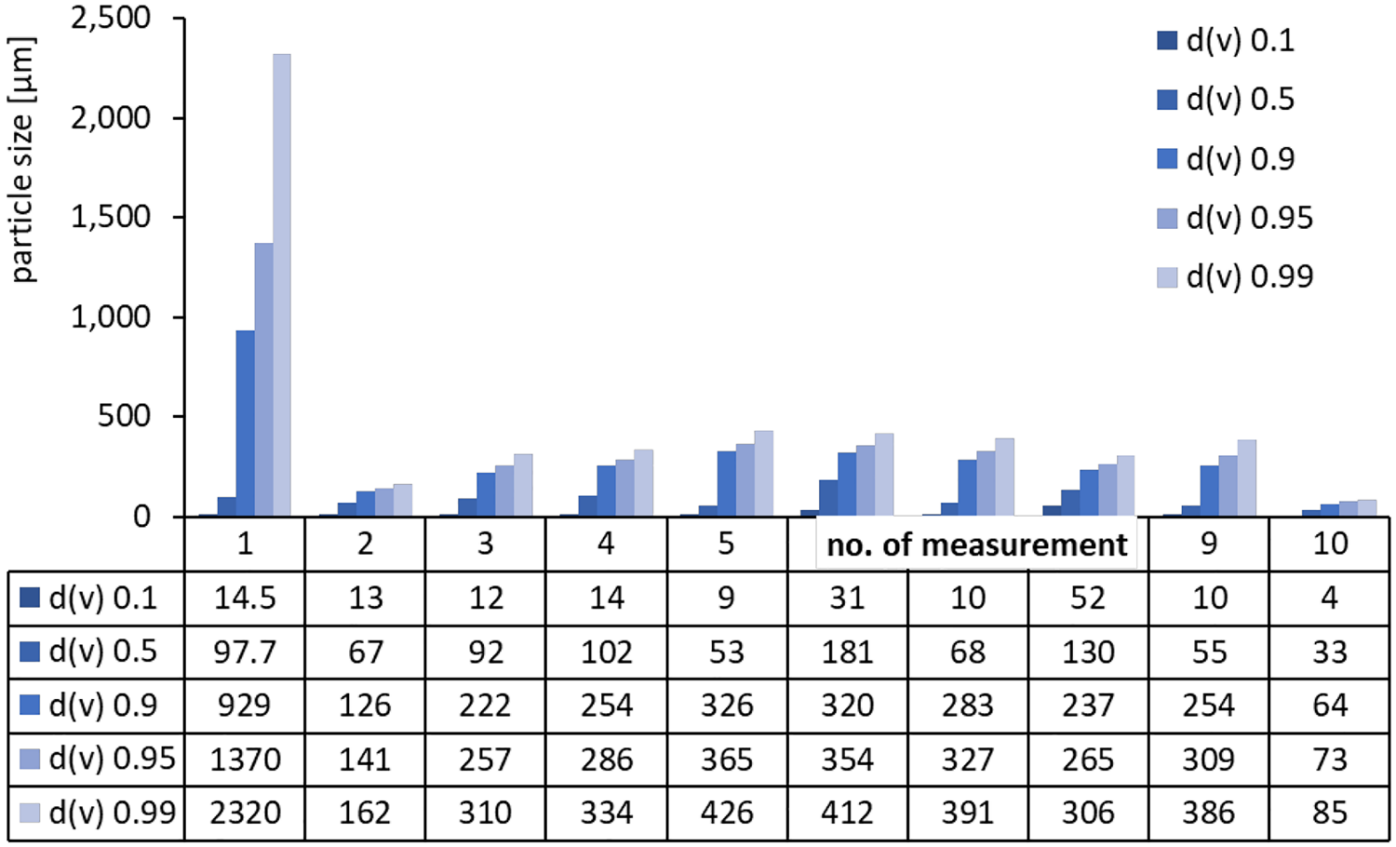
Particle size (LD data) of ethanolic hesperetin solution after addition to water. Data represent the sizes obtained during 10 subsequent measurements

The poor AOC of the ethanolic hesperetin solution was not expected and can only be explained by precipitation of the dissolved molecules upon the addition of the aqueous reaction medium. The concentration of hesperetin used in the assay ranged from 0.66 to 2.67 mmol/L. The solubility of hesperetin is about 73 mmol/L in ethanol but only 2.4 µmol/L in water [33]. Hence, hesperetin concentrations above the saturation solubility of hesperetin were used. As the DPPH assay is performed in small quantities in a 96-well plate, possible precipitation of active might not be reliably detected in the small vessels of the plate. Therefore, to investigate if precipitation was really taking place, the test setup was simulated in a larger volume. For this the ethanolic hesperetin solution was added to water to yield a mixture containing about 0.1% ethanol, 10 µmol/L hesperetin, and water. The mixture was prepared in-situ by adding the ethanolic mixture to water which was already placed in the small volume dispersion unit of the laser diffractometer. This procedure allowed for the analysis of the particle size of the mixture and thus for the detection of possible larger particles, i.e. precipitated hesperetin crystals in the mixture (Fig. 6).

The results show the formation of large particles with sizes to up to 2.3 mm. The particle size decreases rapidly with ongoing size analysis and results in sizes in the micrometer range after 10 measurements. In fact, the assumption that precipitation of hesperetin is taking place after the ethanolic solution is added to the aqueous test medium could be confirmed by this measurement and the low AOC of the ethanolic hesperetin solution can therefore be explained by this phenomenon.

#### Determination of bioenergetics in an Alzheimer cell culture model

**Fig. 7:**
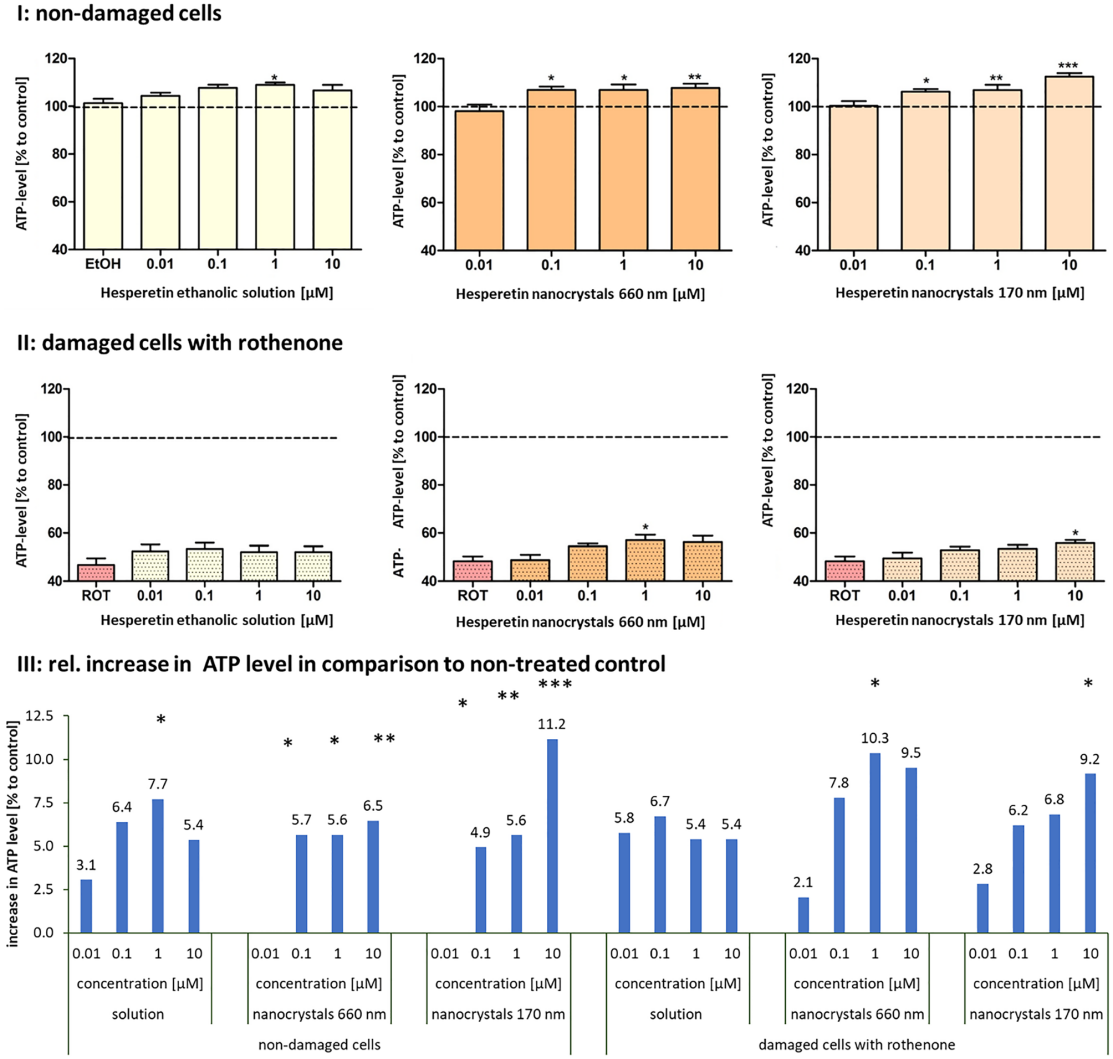
Bioefficacy of hesperetin in an AD cell culture model. Cells were treated with ethanolic hesperetin solution (left), hesperetin nanocrystals 680 nm (middle), or hesperetin nanocrystals 170 nm (0.1–0.3 mg/ml). I: model for early stage of AD-basal ATP levels of SH-SY5Y-AßAPP_wt_ cells after 24 h incubation with different concentrations (0.01–10 µM). II: model for progressed stage of AD-ATP levels of SH-SY5Y-AßAPP_wt_ cells after insult with rotenone (25 µM) for 24 h and after preincubation with the different hesperetin formulations for 1 h and insult with rotenone (25 µM) for 24 h. Cells treated with cell culture medium served as control (100%); cells treated with the respective ethanol concentration did not show significantly altered ATP levels (left); *n* = 8; mean ± SEM; *t* test; ^∗^*p* < 0.05; ^∗∗^*p* < 0.01, ^∗∗∗^*p* < 0.001. III: rel. increase in ATP levels when compared with untreated control

In the last part of the study the formulations were tested in a previously developed cell model of the initial phase of sporadic AD [13]. SH-SY5Y-A-β-PP_wt_ cells produce relatively low levels of neurotoxic Aβ and show impaired mitochondrial functions [13]. To simulate a more progressed stage of AD, cells were additionally treated with rothenone, a specific complex I inhibitor. The hesperetin solution increased ATP levels in a dose-dependent manner. Significant effects were observed at a concentration of 1 µM, and higher doses could not yield a further increase in ATP levels (Fig. 7I—left). The nanocrystal formulations showed no effects at the lowest concentration (0.01 µM), which can be explained by too low amounts of dissolved active. At higher concentrations both nanocrystal formulations showed significant effects on the ATP levels (Fig. 7I—middle and left) and nanocrystals with a size of 170 nm and a concentration of 10 µM increased the ATP level by 13% when compared with the control, which comprises the best effectiveness of all formulations tested (Fig. 7 I and III). Data suggest that hesperetin nanocrystal formulations show enhanced biological effects when compared with the hesperetin solution and indicate that these effects were more pronounced for the small-sized nanocrystals, which possess the highest solubility.

**Fig. 8:**
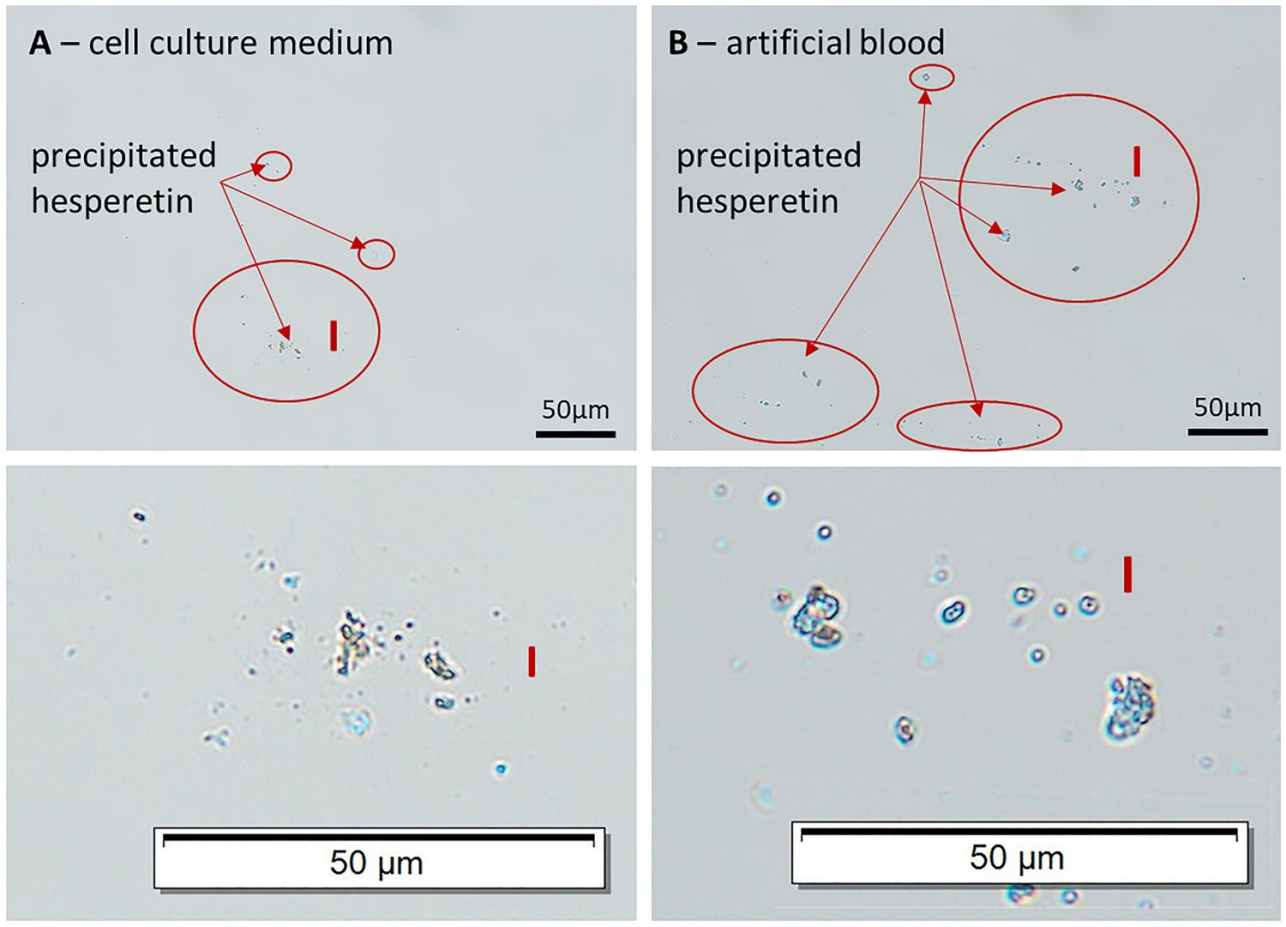
Microscopic images of ethanolic hesperetin solution dispersed in A—cell culture medium, B—artificial blood (0.9% NaCl solution). Upper—original images, Lower—images after digital zooming

However, data obtained are not conclusive, because a solution, where all active is molecularly dissolved, should always lead to the highest biological efficacy. As this was not the case in our study, it was hypothesized that the reduced biological efficacy of the hesperetin solution might be due to a reduction of dissolved active which might be caused by precipitation of hesperetin in the cell culture medium. Therefore, to prove or disprove this theory, a more detailed characterization of the hesperetin solution in cell culture medium was performed. Macroscopic observation did not give any hint for any precipitation of hesperetin. However, microscopic observation revealed the presence of particles with sizes in the range between 1 and 10 µm (Fig. 8, left), thus explaining the non-expected results obtained from this part of the study.

Incubation with rotenone led to decreased ATP levels (50% compared with control) and the treatment with hesperetin solution had no effect on this insult (Fig. 7 II—left). In contrast, both hesperetin nanocrystal formulations increased the ATP levels at 1 µM and 10 µM, respectively (Fig. 7 II—middle and right) and larger-sized nanocrystals were found to be slightly more effective than the smaller-sized nanocrystals (Fig. 7 III). Again, these data were not expected and seemed to be not conclusive at the first glance, because initially it was expected that only dose dependent differences, but no differences between the different formulations would be observed. This was assumed, because it was expected that all active—independent on the formulation used—can be dissolved in the cell culture experiment, thus leading to similar amounts of dissolved active and to similar in vitro effects, therefore. Due to the unexpected results obtained for the ethanolic hesperetin solution, which could be explained by a partial precipitation of the active, also for the other results obtained a more detailed interpretation of the data seemed to be necessary. Hence, it was aimed to investigate if also for the differently sized nanocrystals a reduction of freely dissolved molecules might have occurred during the cell culture tests.

**Fig. 9:**
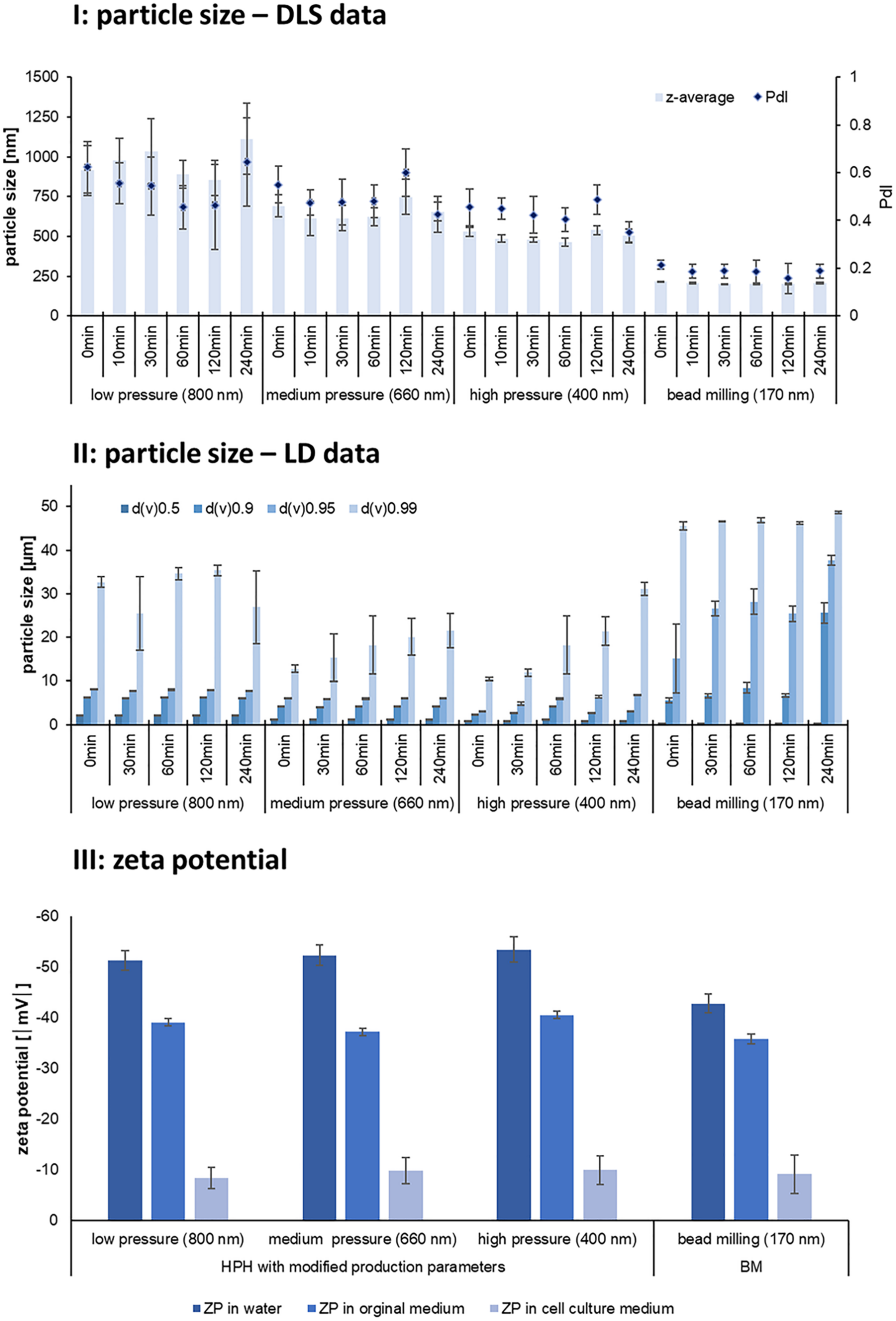
Particle size and zeta potential of hesperetin nanocrystals in cell culture medium. I: PCS data (*z*-average and PdI); II: LD data (median volume diameters (*d*(*v*)0.5–*d*(*v*)0.99), III: Determination of zeta potentials in cell culture medium and comparison to ZP in water and original dispersion medium

In case of nanocrystals a reduction in solubility is possible due to re-crystallisation and/or due to agglomeration of particles. Both phenomena result in the formation of larger particles and thus will lead to a reduction in kinetic solubility and dissolution velocity (c.f. 3.2). Therefore, to investigate if larger particles were formed during the cell culture experiments, the cell culture experiments were simulated by adding the formulations to cell culture media (without cells). Possible changes in size were monitored over time by analysing the size by both, dynamic laser light scattering and by laser diffraction (Fig. 9 I and II). In addition, the ZP of the particles was analysed in cell culture medium and compared with the ZP obtained in water and original dispersion medium (Fig. 9 III).

Size analysis revealed no changes in size for all nanocrystals when analysed with dynamic light scattering (Fig. 9 I). However, when looking at the sizes obtained by laser diffraction (Fig. 9 II), clear changes in size become obvious. Changes in size were not detected for the d(v)0.5 values. Hence, data confirm results obtained from the PCS analysis and prove that the mean size was not affected by the addition of the cell culture medium. However, significant changes were detected for the *d*(*v*)0.99 values. Hence, in all formulations about 1% of the volume of the particles processed sizes between 20 and 50 µm. The *d*(*v*)0.9 and *d*(*v*)0.95 values of the larger-sized nanocrystals, that were produced by HPH, were not affected by the addition of the cell culture media. Hence, the particle sizes remained unchanged and did not show any particle growth. In contrast, the *d*(v)0.9 and *d*(*v*)0.95 values of the small-sized nanocrystals, that were produced by bead milling, were strongly affected by the addition of the cell culture medium and resulted in *d*(*v*)0.9 values > 25 µm. This means that in contrast to the larger-sized nanocrystals, where only < 1% of the particles possessed sizes > 25 µm, the small-sized nanocrystals contained at least 10% of undissolved hesperetin, i.e. hesperetin microcrystals, which possess low solubility and dissolution velocity (c.f. 3.2.). Hence, in the cell culture experiments, less dissolved active was available in the initially small-sized nanocrystal formulation (170 nm) than for the larger-sized nanocrystals (660 nm). Thus, the differences in efficacy, which were not conclusive at the beginning, become logical by taking the changes in size upon the addition of the cell culture medium into consideration.

**Fig. 10:**
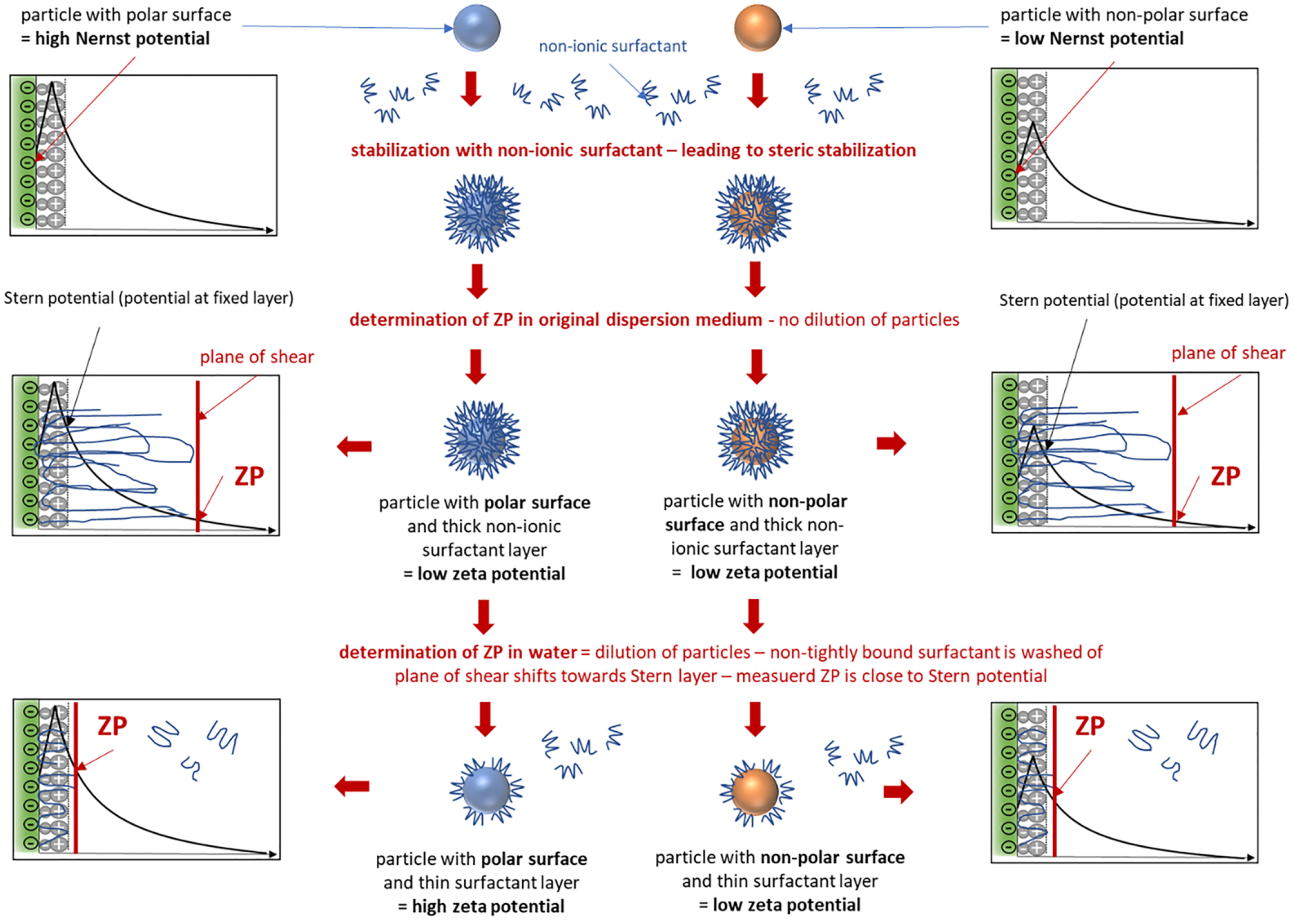
Scheme of zeta potential analysis of nanoparticles in different dispersion media. Upon addition of a non-ionic stabilizer, a thick polymer layer is formed around the particles. Analysis in original dispersion medium analyses the charge of the particles in original state. Analysis in water analyses the charge close to the Stern layer (fixed layer). As the Stern potential is closely linked to the Nernst potential, differences in surface polarity of the particles can be discriminated

Reasons for the more pronounced increase in size for the small-sized nanocrystals can be explained by the production method and the resulting differences in zeta potentials. Only the small-sized nanocrystals were produced by bead milling, whereas all larger-sized formulations were produced by HPH. Zeta potential analysis in water revealed lower ZP values for the nanocrystals that were produced by bead milling, whereas no differences in ZP were found when the ZP was analysed in original dispersion medium or cell culture medium (Fig. 9 III). Zeta potential analysis in cell culture media was performed to determine the influence of the cell culture medium on the physical stability of the particles. Results revealed a strong decrease in ZP for all formulation to < |10| mV and thus a similarly strong impact of the cell culture medium on the physical stability of the particles. Hence, from this set of data the differences in physical stability cannot be explained. Also, no differences in ZP were obtained when the particles were analysed in original dispersion medium, which indicates that the surfactant layer around the particles is similar for both particles. However, the differences in ZP analysed in water give a clear hint that the small-sized nanocrystals (170 nm) possess a more hydrophobic (less polar) surface than the nanocrystals that were produced by HPH. Because ZP analysis in water means that the particles are diluted for the measurement, it means that the surrounding surfactant layer, that is not tightly bound to the surface of the particles, is washed off, thus leading to the determination of the Stern Potential, which is strongly related to the Nernst potential of the particles [37], (Fig. 10). Based on this observation it can be assumed that dilution with cell culture medium—which will also lead to this wash off effect and a subsequent rearrangement of the surfactant layer—will cause a more pronounced agglomeration of the small-sized particles with hydrophobic surface than for the particles with a more hydrophilic surface. This is because the interfacial free energy is higher for the more hydrophobic surface, and thus, also the tendency to reduce the total surface area is higher, which in consequence, leads to a more pronounced agglomeration and increase in size.

Based on the observations that both—ethanolic hesperetin solution and nanocrystals—changed their physicochemical properties during the cell culture experiments, it needs to be concluded that the real potential of hesperetin for the prevention and treatment of AD could not fully be exploited in this study. Nonetheless, results demonstrate the great potential of this active for the treatment of AD.

Results also demonstrate the urgent need to characterize the physico-chemical properties of all samples that are used in the biological test assays not only in-vitro but also in the biological test media itself. Data show that the classical physical-chemical testing is not bio-predictive and thus can only be used for quality control of the formulations. Testing the physical-stability and dissolution of the formulations in cell culture medium is at least in-vitro predictive. However, it can still not provide a complete in-vivo-predictivity. Solutions of active compounds in organic solvent are highly likely to precipitate in the aqueous cell culture media, especially if high concentrations and/or poorly water-soluble actives are used. As seen in our study, it is very likely that the precipitate cannot be seen by naked eye, because only micro-precipitates with sizes in the micrometer range are formed. As only particles with sizes >  150 µm are regarded to be visible, smaller-sized, non-visible particles can only be detected by using for example light microscopy. Hence, based on our experience, whenever organic solutions of actives are used in cell culture tests, light microscopy should be used to prove that the API does not precipitate in the cell culture test. The same is recommended when organic solutions are tested in other biological media, because also here precipitations are very likely to occur if the solubility of the active is not sufficiently high in the biological medium. In case blood is the test medium, a simple test would be the dispersion of the solution in artificial blood, i.e. isotonic NaCl solution. Due to the clear appearance of the NaCl solution the formation of crystals is easily detectable (Fig. 8, right). If such possible changes in the formulations are not considered, the interpretation of the biological test results can be misleading and thus lead to false conclusions.

In our study not only the organic solution was found to undergo changes in size but also the nanocrystals were shown to agglomerate to some extent. The agglomerates were not detected when DLS measurements were performed as stand-alone technique for the characterization of the particles and only the use of an additional technique, i.e. laser diffraction, could reliably detect the changes in size. Hence, data demonstrate again, that size analysis is only meaningful if different and independent methods are used [24, 26]. In this way, in our study size characterization of the formulations enabled a meaningful explanation of the results obtained. However, lessons to be learned for future studies are that formulation development should not only include the determination of the physical stability at storage conditions but should also include the testing of the physical stability in biological test media. Consequently, only the formulations being physically stable in the biological test medium should be used for all biological experiments. The optimal formulation will of course possess both physical stability during storage and sufficient physical stability in the biological test setup.

## Conclusions

In this study, hesperetin nanocrystals with sizes between 170 and 800 nm were produced by HPH or BM. Their physical-chemical properties and the antioxidant capacity were determined in-vitro, and the treatment efficacy against Alzheimer’s disease was investigated in a cell culture model, where an organic hesperetin solution served as control. Our study showed that smaller-sized particles (< 200 nm) with a more hydrophobic surface could be obtained by BM, whereas larger-sized nanocrystals (> 300 nm) with a more hydrophilic surface were obtained by HPH. The dissolution velocity, the kinetic solubility, and the AOC, determined in-vitro and in-vivo, increased with decreasing size. However, a pronounced increase in these parameters was only observed for the nanocrystals with sizes < 200 nm. In addition, contrary to the expectations, nanocrystals also possessed a higher AOC than the hesperetin solution. Reasons for this were a partial re-crystallization of hesperetin from the ethanolic solution in the aqueous test medium, which led to a reduced amount of freely dissolved hesperetin molecules und thus to a lower AOC. Precipitation of hesperetin from the ethanolic solutions also occurred in the cell culture experiments. However, only at higher concentrations, the precipitation could not be observed macroscopically. Only microscopic analysis—which is not regularly performed in cell culture experiments—could discover the partial precipitation of the poorly water soluble active and could therefore explain the real reason for the decreased efficacy of the hesperetin solution at higher doses. Also, the physical stability of the hesperetin nanocrystals was found to be impaired by the cell culture medium, which resulted in a partial formation of larger agglomerates. The agglomeration was more pronounced for the small-sized nanocrystals (170 nm—10% agglomerates) than for the larger-sized nanocrystals (660 nm—1% agglomerates). Despite agglomeration, addition of the hesperetin nanocrystals to SH-SY5Y AßPP_wt_ cells led to a significant increase in ATP levels, which was more pronounced for the small-sized nanocrystals. Hence, to exploit the full potential of nanocrystals, i.e. increased dissolution velocity, kinetic solubility, and biological activity, small-sized nanocrystals with sizes < 200 nm should be produced.

## Data Availability

All datasets generated are available on request.
